# Cardiac Metabolism and Contractile Function in Mice with Reduced Trans-Endothelial Fatty Acid Transport

**DOI:** 10.3390/metabo11120889

**Published:** 2021-12-19

**Authors:** Tatsuya Iso, Masahiko Kurabayashi

**Affiliations:** 1 Department of Medical Technology and Clinical Engineering, Faculty of Medical Technology and Clinical Engineering, Gunma University of Health and Welfare, 191-1 Kawamagari-Machi, Maebashi 371-0823, Gunma, Japan; 2 Department of Cardiovascular Medicine, Gunma University Graduate School of Medicine, 3-39-22 Showa-Machi, Maebashi 371-8511, Gunma, Japan; mkuraba@gunma-u.ac.jp

**Keywords:** cardiac metabolism, fatty acid, glucose, capillary endothelium, trans-endothelial fatty acid transport, TCA cycle, pool size, contractile function

## Abstract

The heart is a metabolic omnivore that combusts a considerable amount of energy substrates, mainly long-chain fatty acids (FAs) and others such as glucose, lactate, ketone bodies, and amino acids. There is emerging evidence that muscle-type continuous capillaries comprise the rate-limiting barrier that regulates FA uptake into cardiomyocytes. The transport of FAs across the capillary endothelium is composed of three major steps—the lipolysis of triglyceride on the luminal side of the endothelium, FA uptake by the plasma membrane, and intracellular FA transport by cytosolic proteins. In the heart, impaired trans-endothelial FA (TEFA) transport causes reduced FA uptake, with a compensatory increase in glucose use. In most cases, mice with reduced FA uptake exhibit preserved cardiac function under unstressed conditions. When the workload is increased, however, the total energy supply relative to its demand (estimated with pool size in the tricarboxylic acid (TCA) cycle) is significantly diminished, resulting in contractile dysfunction. The supplementation of alternative fuels, such as medium-chain FAs and ketone bodies, at least partially restores contractile dysfunction, indicating that energy insufficiency due to reduced FA supply is the predominant cause of cardiac dysfunction. Based on recent in vivo findings, this review provides the following information related to TEFA transport: (1) the mechanisms of FA uptake by the heart, including TEFA transport; (2) the molecular mechanisms underlying the induction of genes associated with TEFA transport; (3) in vivo cardiac metabolism and contractile function in mice with reduced TEFA transport under unstressed conditions; and (4) in vivo contractile dysfunction in mice with reduced TEFA transport under diseased conditions, including an increased afterload and streptozotocin-induced diabetes.

## 1. Introduction

The heart is well-known as a metabolic omnivore that selects energy substrates suitable for physiological and pathophysiological circumstances [1,2,3,4]. It is capable of consuming fatty acids (FAs), glucose, lactate, ketone bodies, acetate, and amino acids [1,2,3,4,5]. In the adult heart, preference for these substrates is dynamically changed by substrate availability, hormones, oxygen supply, and cardiac workload. Hearts with pathological hypertrophy, which revert to a fetal metabolic profile, rely on more glucose with a reduced capacity of FA oxidation (FAO). In addition to adenosine triphosphate (ATP) synthesis (catabolism), energy substrates, especially glucose, are linked to the facilitation of anabolic and accessory pathways depending on the energy status and the pathophysiological situation. Synthesized ATP is mainly used for mechanical contraction, Ca^2+^ uptake into the sarcoplasmic reticulum, and the maintenance of the sarcolemmal ion gradients. Under normal conditions, nearly all ATP is generated from mitochondrial oxidation; 2% or less are derived from anaerobic glycolysis. FAO supplies 60–90% of myocardial ATP in a healthy adult heart, whereas the remaining (10–40%) comes from glucose, lactate, ketone bodies, acetate, and certain amino acids, such as branched-chain amino acids and glutamine [1,2,3,4,5]. Because the myocardial ATP pool is limited, the heart needs to constantly generate ATP by catabolizing substrates in response to energy demands.

The capillary endothelium is a crucial blood–tissue interface that controls the energy supply according to the organs’ needs [6,7,8,9]. Histologically, there are three major types of capillaries—(1) continuous and non-fenestrated capillaries that permit the paracellular passive diffusion of water and small solutes but not larger molecules (i.e., the brain–blood barrier and muscle-type continuous capillaries); (2) fenestrated capillaries with intracellular pores that contain diaphragms that are found in several endocrine organs, glomeruli, and the gut; and (3) sinusoidal discontinuous capillaries with intercellular gaps that are found in the liver, spleen, bone marrow, and some endocrine organs [6]. Among them, there is emerging evidence that muscle-type continuous capillaries play an important role in the transfer of nutrients and hormones to parenchymal cells [6,7,8,9]. In fat-utilizing tissues, such as the heart, skeletal muscle, and adipose tissue, long-chain FAs are transported to the interstitial space through the muscle-type capillary endothelium by a mechanism composed of three major steps—the lipolysis of triglyceride (TG) on the luminal side of the endothelium, FA uptake by the plasma membrane, and intracellular FA transport by cytosolic proteins. Because trans-endothelial FA (TEFA) transport is the first rate-limiting step in the utilization of parenchymal cells, its impairment causes dyslipidemia and reduced FA uptake in peripheral tissues, including the heart, skeletal muscle, and adipose tissue. In the heart, the defective function of TEFA transport frequently exerts reduced FA uptake, with a dramatic increase in glucose utilization and a decline in contractile function under normal and diseased conditions because the heart heavily relies on FA combustion. Accordingly, this review is focused on four main points: (1) the mechanisms of FA uptake by the heart, including TEFA transport; (2) molecular mechanisms underlying the induction of genes associated with TEFA transport; (3) in vivo cardiac metabolism and contractile function in mice with reduced TEFA transport under unstressed conditions; and (4) in vivo contractile dysfunction in mice with reduced TEFA transport under diseased conditions, including an increased afterload and streptozotocin-induced diabetes.

## 2. Mechanisms of FA Uptake by the Heart

### 2.1. Source of Long-Chain Fatty Acids

**Figure 1:**
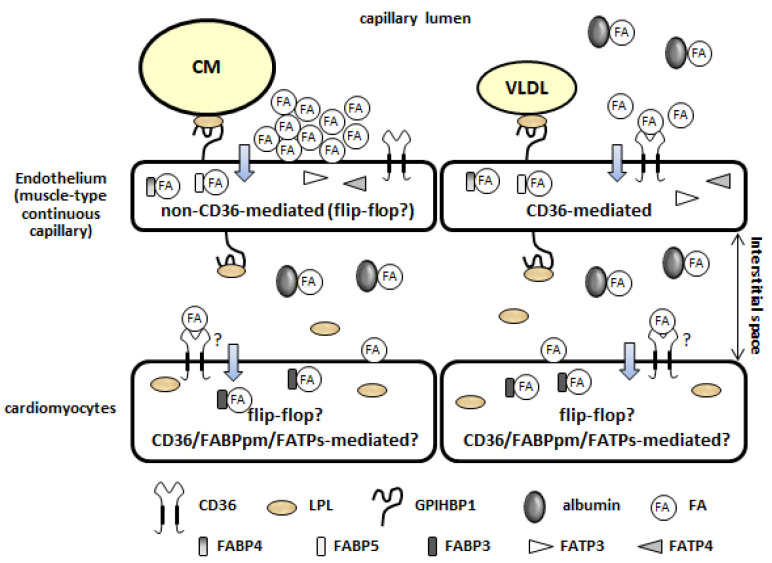
Mechanisms of fatty acid uptake by the heart. (1) Lipolysis of TG contained in TGRLPs on the luminal side of the capillary endothelium; (2) FA uptake by the plasma membrane of the capillary endothelium; (3) intracellular FA transport through the capillary endothelium; (4) FA uptake by cardiomyocytes.

As shown in Figure 1, FAs are supplied to the heart as either free FAs (FFAs) bound to albumin or as FAs released from the TG contained in TG-rich lipoproteins (TGRLPs): chylomicrons (CM) that are synthesized in the intestine from exogenous dietary fat and very low-density lipoproteins (VLDL) that are synthesized by the liver from endogenous lipids [10,11,12,13,14]. FFAs bound to albumin originate from adipose tissue lipolysis, with some derived from “spillover” through the action of lipoprotein lipase (LPL). Both circulating FFAs and TGRLPs significantly contribute to the overall FA supply to cardiomyocytes. Normal circulating FFA concentrations range between 0.2 and 0.6 mM. These levels can dramatically vary from very low concentrations in fetal circulation to over 2 mM during severe stress, such as myocardial ischemia and uncontrolled diabetes. Prolonged fasting also increases the release of FFAs from adipose tissue. Chronic or acute increases in circulating FFAs have major impacts on the rates of cardiac FA uptake and β-oxidation, as arterial FA concentration is the primary determinant of the rate of myocardial FA uptake and oxidation. Plasma TG concentration is also highly variable—fasting plasma TG is typically 0.6–0.7 mM, increasing to 1.5–3.0 mM following a mixed meal [15]. Because TG yields three times the FAs upon complete hydrolysis, the availability of plasma TG-FA greatly (90%) exceeds the availability of circulating FFAs. However, defining the myocardial preference for lipids, albumin-bound FFAs, or FAs from TGRLPs has proven difficult [12].

### 2.2. Lipolysis of TG Contained in TG-Rich Lipoproteins on the Luminal Side of the Capillary Endothelium

LPL is an essential enzyme that hydrolyses the TG contained in TGRLPs [10,11,12,13]. Importantly, LPL is predominantly produced in cardiomyocytes and is transferred to the luminal side of the endothelium, where the enzyme functions (Figure 1). GPIHBP1, a glycosylphosphatidylinositol-anchored protein 1 expressed in the capillary endothelium, is the principal binding site for LPL on the endothelium (Figure 1). GPIHBP1 binds to LPL from interstitial spaces and shuttles it across the endothelium to the capillary lumen. On the luminal side, its ability to bind to both LPL and TGRLPs allows it to serve as a platform for TG lipolysis [11,16]. The VLDL receptor, expressed in the capillary endothelium, functions as a peripheral receptor for TGRLPs and facilitates the hydrolysis of TG in concert with LPL [12,17,18].

### 2.3. Fatty Acid Uptake by the Plasma Membrane of the Capillary Endothelium (Non-CD36-Mediated and CD36-Mediated Pathways)

There are two distinct pathways of FA uptake by the capillary endothelium [19,20]—a high-capacity non-saturable pathway (Figure 1, upper left) and a low-capacity saturable pathway (Figure 1, upper right). The non-saturable pathway operates at high ratios of FAs. CM-derived TG-FAs (high local release of FA) enter through a non-CD36-mediated route (low affinity, high capacity, and non-saturable, presumably via the flip-flop mechanism) [20]. The saturable pathway has kinetics that are consistent with protein facilitation, with a high affinity for long-chain FAs (Km of approximately 10 nM). CD36, also known as fatty acid translocase (FAT), is a high-affinity receptor for long-chain FAs (Km of 5–10 nM) and is suitable for the low levels of FFAs. Importantly, in the heart, CD36 is more abundant in the capillary endothelium compared to cardiomyocytes [21,22]. It is likely that VLDL-derived TG-FAs (low local release of FAs) and albumin-bound FFAs enter the cell through a CD36-mediated channel (high affinity, low capacity, and saturable).

### 2.4. Intracellular Fatty Acid Transport through the Capillary Endothelium

Following FA uptake via the plasma membrane, intracellular FA transport is performed by cytosolic proteins. Fatty acid-binding proteins 4 and 5 (FABP4/5), abundantly expressed in the capillary endothelium in the heart, are potential candidates for transport (Figure 1) [23,24,25]. Cytoplasmic FABPs (FABP1–FABP9) are a family of 14–15 kDa proteins that bind to long-chain FAs with high affinity. Among them, FABP4/5 have a redundant function in the capillary endothelium. As lipid chaperones, FABP4/5 appear to facilitate the intracellular FA transport to the abluminal side of the capillary endothelium. Fatty acid transport proteins 3 and 4 (FATP3/4), which are induced in the capillary endothelium in response to an increase in vascular endothelial growth factor-B (VEGF-B) secreted from cardiomyocytes, are other candidates for intracellular FA transport (Figure 1) [26]. The facilitation of FA uptake by FATPs seems to be mediated, at least in part, by mitochondrial ATP production and ATP-dependent acyl-CoA synthetase activity [27]. The mechanisms underlying the release of FAs into the interstitial space remain to be determined.

### 2.5. Fatty Acid Uptake by Cardiomyocytes

Following TEFA transport (lipolysis, FA uptake by the plasma membrane, and intracellular FA transport), FAs are bound by albumin (300 μM) in the interstitial space of the heart (Figure 1) [14,28]. Circulating albumin is internalized by fluid-phase uptake by the capillary endothelium and transferred to the interstitial space by transcytosis [29,30]. The trans-sarcolemmal uptake of FA by cardiomyocytes may be facilitated by membrane-associated proteins. Similar to the capillary endothelium, the main membrane-associated protein might be CD36 in cardiomyocytes (Figure 1), although the expression of CD36 in cardiomyocytes is much lower than that in the capillary endothelium [21,22,31]. Other candidates of membrane-associated proteins are the plasma membrane FABP (FABPpm, also known as GOT2) and FATP1 [31]. However, the contributions of FABPpm and FATP1 to FA uptake in vivo remain to be determined. After being taken up by cardiomyocytes, FAs are bound by cytoplasmic FABP3 (also designated as H-FABP; 150–300 μM), which acts as the intracellular counterpart of albumin [31]. The total FA concentration is 100–400 μM in the interstitial space, whereas it is 50 μM in the cytoplasm. Albumin and FABP3 each provide a buffer for FAs, whereby each FA molecule is immediately replenished by the release of another FA molecule from the proteins. The direction and overall rate of FA uptake are determined by the trans-sarcolemmal gradient of FAs.

## 3. Molecular Mechanisms Underlying the Induction of Genes Associated with Trans-Endothelial Fatty Acid Transport

Recent studies have revealed that the expression of genes associated with TEFA transport is regulated by several ligands, receptors, and transcription factors (Table 1) [6,7,8,9]. It is likely that these systems can be roughly divided into two groups according to their target genes. One includes the peroxisome proliferator-activated receptor γ (PPARγ), mesodermal homeobox-2/transcription factor 15 (Meox2/Tcf15), Notch signaling, and the apelin/apelin receptor (APLNR), and it mainly controls the expression of CD36, FABPs, and GPIHBP1. The other is a group that includes the VEGF-B/VEGF receptor (VEGFR), angiopoietin-like 2 (ANGPTL2), and 3-hydroxyisobutyrate (3-HIB), and it regulates the expression/function of FATP3/4 (Table 1). Although impairments of the systems influence both local and systemic metabolism, cardiac metabolism seems to only be affected by PPARγ, Meox2/Tcf15, Notch signaling, and VEGF-B/VEGFR (Table 1) [6,7,8,9]. The trans-endothelial transport of other substrates and molecules (e.g., lipoproteins, lipoprotein lipase, glucose, and insulin) and endothelium-derived metabolic regulators (e.g., nitric oxide, extracellular matrix proteins, hormones, growth factors, and enzymes) is described elsewhere [6,7,9].

### 3.1. Peroxisome Proliferator-Activated Receptor γ

Cardiac metabolism is transcriptionally regulated by the three members of the PPAR family (PPARα, β/δ, and γ) of ligand-activated transcription factors [32,33,34]. PPARs do not act on a single target but rather orchestrate several pathways whereby nutrients regulate their own metabolisms. The expression of PPARα is high in cardiomyocytes and regulates FA catabolism, such as FA uptake and FA oxidation. PPARγ and its target genes are abundantly expressed in the capillary endothelium in the heart and facilitate FA uptake through the endothelial layer.

The expression of PPARγ is induced in the capillary endothelium by fasting, leading to the induction of its target genes, such as CD36, FABP4, and GPIHBP1 [35,36,37]. Endothelial-specific PPARγ knockout mice exhibited hyperchylomicronemia after olive oil gavage and higher levels of circulating FFAs during fasting, results that are consistent with the defective function of GPIHBP1 (via LPL) and CD36/FABP4, respectively [35,36]. Thus, endothelial PPARγ in the heart facilitates FA uptake via both an LPL-mediated low-affinity, high-capacity, non-saturable pathway and a CD36-mediated high-affinity, low-capacity, saturable pathway, both of which are enhanced during fasting.

**Table 1:** FA handling genes regulated by the indicated system in capillary endothelium.

Ligand	Receptor/Transcription Factor	Target Genes										Target Tissues Influenced by the System	Reference
		PPARγ	CD36	FABP4	FABP5	LPL	GPIHBP1	ANGPTL4	LIPG	FATP3	FATP4		
	PPARγ		⚪	⚪			⚪					heart, skeletal muscle, adipose tissue	[35,36,37]
	Meox2/Tcf15	⚪	⚪	⚪	⚪	⚪	⚪					heart	[38]
Dll4	Notch1/N1-ICD/Rbp-jκ	independent	⚪	⚪	⚪			⚫	⚪			heart, skeletal muscle	[39,40]
Apelin	APLNR/phosphorylation of FOXO1			⚫								skeletal muscle	[41]
VEGF-B	VEGFR/NPR1									⚪	⚪	heart, BAT, skeletal muscle	[26]
ANGPTL2	integrin α5β1		⚪							⚪		subcutaneous adipose tissue	[42]
3-HIB										⚪*	⚪*	skeletal muscle	[43]

⚪ induced; ⚫ suppressed; ⚪* post-translational effect?

### 3.2. Mesodermal Homeobox-2/Transcription Factor 15

Meox2 is a homeobox gene that is expressed in the microvascular endothelium in the heart [38]. Meox2 forms a heterodimer with a basic helix–loop–helix Tcf15, which is highly expressed in the capillary endothelium. The Meox2/Tcf15 heterodimer drives the endothelial expression of genes associated with FA metabolism, including PPARγ, CD36, FABP4/5, LPL, and GPIHBP1, to facilitate FA uptake and transport across the capillary endothelium [38]. Importantly, the haplodeficiency of Meox2/Tcf15 in mice was found to cause reduced FA uptake with compensatory glucose use in the heart (Table 2), suggesting their robust effects on cardiac metabolism.

### 3.3. Notch Signaling

Notch signaling is not only a master regulator of angiogenesis but also a regulator of TEFA transport. The inhibition of endothelial Notch signaling in the adult heart leads to reduced FA transport, resulting in heart failure and hypertrophy [39,40]. The activation of Notch signaling facilitates FA transport by inducing CD36, FABP4/5, and lipase G endothelial type (LIPG) and by suppressing angiopoietin-like 4 (ANGPTL4), a well-characterized inhibitor of LPL [39,40].

**Table 2:** Cardiac metabolism and performance in vivo in the indicated knockout mice under unstressed condition.

Target Genes	Deficient Site	Inducible Knockout	VLDL-TG Uptake	FA Uptake	Glucose Uptake	Glut1/4	Ketonein Serum	Contractile Performance In Vivo Estimated by Echocardiography	Reference
LPL (functions at luminal side of capillary)	cardiomyocyte		↓	↑	↑	↑		↓ aged	[44]
	cardiomyocyte	⚪						↓	[45]
CD36	whole			↓	↑	↑	↑	intact	[46,47,48,49]
	whole			↓	↑			prevention from age-induced cardiomyopathy	[49]
	endothelium			↓	↑	↑		not available	[21]
FABP4/5	whole			↓	↑	↑	↑	intact	[23,50]
Meox2^+/−^:Tcf15^+/−^	endothelium: whole			↓	↑			↓ aged	[38]
Rbp-jκ (Notch signal)	endothelium	⚪		↓	↑	↓		↓↓	[39]
PPARγ	endothelium			→↓	→			intact (personal observation)	[35]
VEGF-B	whole			↓	↑	↑		not available	[26]
FABP3	whole			↓	↑	→	↑	not available	[51,52]
CD36	cardiomyocyte			→	→			not available	[21]
	cardiomyocyte	⚪		↓ (ex vivo)	↑ (ex vivo)			intact	[53,54]

⚪ inducible knockout; ↓ reduced; ↑ increased; → no change.

### 3.4. Apelin/Apelin Receptor/Forkhead Box O1

Apelin is a peptide identified as a ligand of the G protein-coupled receptor APLNR [55]. Apelin/APLNR may be involved in many physiological processes, including angiogenesis, the regulation of blood pressure, and energy metabolism [55]. The endothelial-specific deletion of APLNR enhances TEFA transport via the induction of FABP4, resulting in ectopic lipid deposition in muscle and impaired glucose utilization [41]. APLNR-mediated forkhead box O1 (FOXO1) phosphorylation inactivates its transcriptional activity on FABP4. Thus, Apelin/APLNR is a negative regulator of TEFA transport in skeletal muscle.

### 3.5. Vascular Endothelial Growth Factor-B/Vascular Endothelial Growth Factor Receptor/Fatty Acid Transport Proteins

Paracrine signaling by VEGF-B from peripheral tissues also regulates TEFA transport [26]. VEGF-B is expressed in most adult tissues, with the highest expression found in the myocardium, skeletal muscle, and brown adipose tissue. VEGF-B secreted from myocardium specifically binds to VEGF receptor-1 and the common co-receptor neuropilin-1 expressed in capillary endothelium, which consequently induces the expression of two FATPs, FATP3 and FATP4. Endothelial FATP3/4 are required for FA transport across the vascular endothelial layer. In sharp contrast to the induction of PPARγ target genes during fasting, the expression level of VEGF-B was found to be suppressed by fasting and enhanced by a high-fat diet. These findings suggest that the VEGF-B/VEGFR/FATPs axis and the endothelial PPARγ axis function in different nutritional states. Notably, the findings regarding VEGF-B have not been reproduced by another group [56,57]. Thus, the metabolic effects of VEGF-B signaling need to be re-evaluated in further investigations.

### 3.6. Angiopoietin-Like 2/Integrin α5β1

ANGPTL2 is secreted from adipose tissue [42]. ANGPTL2/integrin α5β1 signaling activates FA transport into subcutaneous adipose tissue via the induction of CD36 and FATP3 in the capillary endothelium, which suggests adipocyte–endothelial crosstalk.

### 3.7. 3-Hydroxyisobutyrate

3-HIB is a catabolic intermediate of a branched-chain amino acid valine and is secreted from skeletal muscle [43]. 3-HIB has been found to regulate TEFA transport in a paracrine fashion, probably via a post-translational effect on FATP3 and FATP4. Increased 3-HIB was found to promote lipid accumulation in muscle, leading to insulin resistance. This is the first reported evidence that metabolites can also modulate TEFA transport.

## 4. Association between In Vivo Cardiac Metabolism and Contractile Function in Mice with Reduced Fatty Acid Uptake

### 4.1. Limitation of Experiments with Ex Vivo Perfused Hearts

Heart metabolism has long been studied, primarily in ex vivo perfused hearts. The major benefits of this approach include the ability to simultaneously monitor the oxidation of energy substrates (catabolism) and control hemodynamic parameters, which have provided invaluable assets in the metabolic research of the heart [2]. However, this approach has a weakness in addressing questions related to the metabolic disturbance of the heart in the context of oxygen supply and systemic response because isolated perfused hearts have a lower oxygen-carrying capacity, regional anoxia due to arteriole constriction, a lack of compensatory energy supply from blood, and a lack of neurohumoral feedback [58,59,60]. Moreover, few studies with ex vivo and in vivo models have simultaneously estimated catabolic and anabolic pathways that are invariably affected during the progression of heart failure. The focus of this section is on the association between cardiac metabolism and contractile function investigated by in vivo experiments in mice—an association that has been insufficiently addressed. In vivo cardiac metabolism is mainly estimated by the biodistribution of glucose/FA analogs labeled with radioactive isotopes, the measurement of water-soluble metabolites by mass-spectrometry-based metabolome analysis, and metabolic flux analysis with stable isotopes.

### 4.2. In Vivo Cardiac Metabolism and Contractile Function in Mice with Reduced Trans-Endothelial Fatty Acid Transport under Unstressed Conditions

Various phenotypic changes in metabolism have been reported in both humans and mice when FA catabolism is genetically disrupted. Defective FA oxidation at a mitochondrial level leads to severe impairments in local and systemic metabolism, including hypoketotic hypoglycemia, liver dysfunction, myopathy/rhabdomyolysis, arrhythmia, and cardiomyopathy, which frequently causes sudden infant death syndrome in humans [61,62]. In comparison, reduced FA uptake without defective machinery for mitochondrial FA oxidation results in a modest metabolic phenotype. In most cases, animals with this condition are born and normally develop, although they experience local and systemic alterations in their metabolism. Table 2 summarizes the metabolic and cardiac phenotypes of mice with reduced FA uptake, mostly from those with impaired TEFA transport. Table 2 also includes the phenotypes of whole CD36 KO mice, cardiomyocyte-specific CD36 KO mice, and whole FABP3 KO mice for comparison with impaired TEFA transport.

In general, reduced FA uptake accompanies compensatory glucose use in the heart (Table 2; the mechanism of compensatory glucose uptake is further discussed in Section 4.6). In addition, in the cases of mice with a systemic deletion of FA-handling genes, such as CD36, FABP4/5, and FABP3 [23,46,47,48,51,52], the levels of ketone bodies in the serum and the expression of ketolytic enzymes in the heart have been found to be increased, suggesting both local and systemic responses to energy insufficiency. Moreover, in cardiac-specific LPL KO mice, even FFA uptake (not from TGRLP) was found to be enhanced [44]. These findings suggest that the heart is likely to combust any available energy substrates when the FA supply is limited, which is consistent with the concept that the heart is a metabolic omnivore. Endothelial PPARγ KO mice did not exhibit either reduced FA uptake or elevated glucose use, although they have shown a modest reduction in FA uptake during the postprandial period [35]. No overt reduction in FA uptake in the mice may have been caused by the basal and sufficient expression of PPARγ target genes, even in the absence of its activation. In most mice with a genetic deletion of the genes (Table 2), their contractile function was found to be intact. In contrast, contractile alteration appeared in aged mice and those with an inducible deletion of target genes at adulthood. In aged mice with a cardiac-specific deletion of LPL [44] and Meox2^+/−^:Tcf15^+/−^ [38], cardiac contraction has been found to be reduced, while CD36 KO mice have exhibited the prevention of age-induced cardiomyopathy [49]. The reason for opposite results regarding cardiac contraction is still unclear. The inducible deletion of LPL [45] and Rbp-Jκ [39] results in a reduction in cardiac contractility. The acute suppression of FA uptake machinery by inducible ablation might cause the insufficient induction of systems for compensatory energy use. Indeed, Glut4 mRNA levels were found to be reduced and compensatory glucose uptake was shown to be modest in mice with an inducible deletion of Rbp-Jκ [39], and a ketogenic diet improved contractile dysfunction in the mice [39], indicating that contractile dysfunction is attributed to energy insufficiency. Thus, in most cases of mice with the genetic deletion of genes associated with FA uptake, contractile function is preserved by local and systemic compensation, while it is reduced with inducible deletion due to an insufficient response.

### 4.3. In Vivo Cardiac Metabolism in CD36 KO Mice under Unstressed Conditions

CD36 facilitates FA uptake in the heart, skeletal muscle, and adipose tissue. In the heart, CD36 is more abundant in the capillary endothelium compared to other cell types, including cardiomyocytes [21,22]. Endothelial-specific CD36 KO mice have shown reduced FA uptake with compensatory glucose use in the heart, which recapitulated the metabolic phenotype of whole CD36 KO mice (Table 2) [21,46,47,48]. In contrast, cardiomyocyte-specific CD36 KO mice were shown to exhibit no alteration in the uptake of FA and glucose, although lipid accumulation was reduced in the heart [21]. Another group reported that the inducible deletion of CD36 in cardiomyocytes led to no obvious cardiac dysfunction in vivo, although FA uptake was suppressed, at least in an isolated working heart [53,54]. Taken together, these findings suggest that endothelial CD36 plays a more predominant role in FA uptake in the heart than its expression in cardiomyocytes. It is warranted to study whether the inducible deletion of CD36 in endothelium causes contractile dysfunction.

### 4.4. In Vivo Contractile Dysfunction in Mice with Reduced Trans-Endothelial Fatty Acid Transport under an Increased Afterload

**Figure 2:**
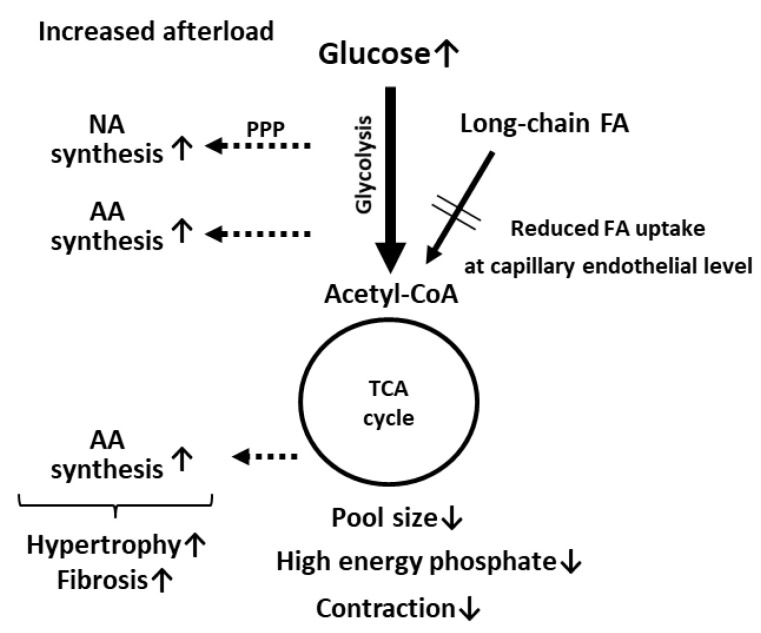
Catabolic pathways were suppressed and anabolic pathways were enhanced in pressure-overloaded hearts in mice with reduced FA uptake and compensatory glucose use. NA, nucleic acids; AA, amino acids; PPP, pentose phosphate pathway.

Cardiac contraction is mostly preserved in mice with a genetic deletion of genes associated with FA uptake, as previously described. However, contractile function is significantly suppressed by an increased afterload in LPL KO [44], CD36 KO [46,47,53], and FABP4/5 double KO (DKO) mice [50], suggesting compromised energetics. Metabolome and metabolic flux analyses performed with CD36 KO and FABP4/5 DKO mice provided two major findings that could account for the link between contractile dysfunction and compromised energetics [47,50]. One prospective finding is a reduction in the pool size (total intermediates) in the TCA cycle (Figure 2). It was reported that a reduced pool size in an isolated working heart results in a decline in contractile function, which is restored by alternative fuels [63]. Pool size also appears to be a useful marker of the energy status in the KO hearts in vivo. Even in the hearts under unstressed conditions, pool size was significantly reduced (Figure 2) [47,50]. The reduction in pool size was further enhanced by an increased afterload (Figure 2). Because pool size was found to more significantly change in comparison to high energy phosphate, pool size could be an alternative marker to assess the energy status of the heart. A complicated relationship between pool size and contractile function is described in detail in Section 4.5. Another intriguing finding of metabolome and metabolic flux analyses is the presence of multiple pathways for glucose use in pressure-overloaded hearts [47,50]. Glucose is used not only for catabolism for ATP synthesis but also for anabolism to generate building blocks for biomass synthesis (e.g., amino acids and nucleic acids) (Figure 2). In KO hearts, the most unexpected finding by flux analysis was the enrichment of glucose-derived glutamate and aspartate [47,50]. In general, enhanced glycolytic flux is tightly coupled with the malate–aspartate shuttle for balancing the cytosolic NAD^+^/NADH redox pair [64,65]. Because glutamate and aspartate are essential components for the malate–aspartate shuttle, it is conceivable that the synthetic rate of glutamate and aspartate from glucose is markedly accelerated in the heart. When glycolysis is further enhanced by an increased afterload, the synthetic rate of glucose-derived glutamate and aspartate is more facilitated [47,50]. Synthesized amino acids from glucose in the heart with an increased workload could serve as building blocks of biomass synthesis for hypertrophic response and increased fibrosis (Figure 2). The pentose phosphate pathway is also likely to be accelerated by an increased afterload in the heart with reduced FA uptake [50], which can lead to the enhanced synthesis of nucleic acids. Thus, glucose is preferentially utilized for biomass synthesis rather than ATP production, which may enhance the diminishment of pool size and resultant contractile dysfunction (Figure 2).

Medium-chain FAs (MCFAs) can bypass CD36 for entry into cardiomyocytes. Feeding mice an MCFA-rich diet was found to improve contractile dysfunction in CD36 KO and FABP4/5 DKO mice [47,50,53], which further suggests that the efficient supplementation of alternative fuels may restore cardiac dysfunction caused by energy insufficiency. FABP3 KO mice were found to be intolerant to endurance exercise (Table 2) [52], which also suggests the heart cannot respond to an increased energy demand. Thus, a heart with reduced FA uptake becomes energetically compromised when the energy demand is enhanced by an increased workload. These findings strongly suggest that a total energy supply that meets the energy demand is crucial for the normal functioning of the heart, rather than the selectivity of energy substrates.

### 4.5. Pool Size in the TCA Cycle as a Useful Marker for Energy Status

**Figure 3:**
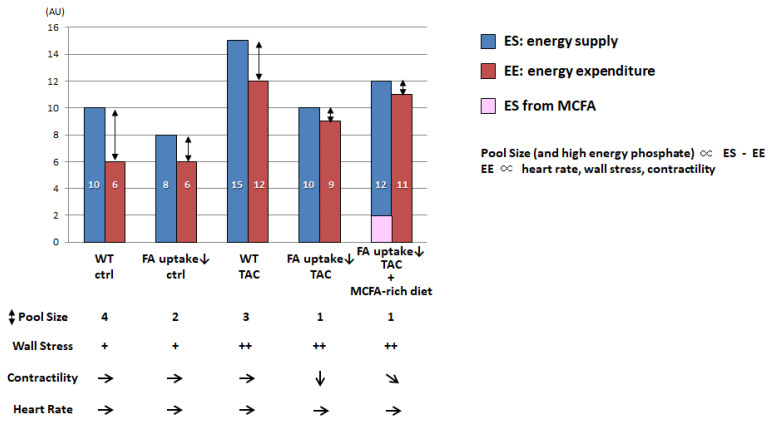
Putative bar graph regarding the pool size of the TCA cycle associated with the difference between energy supply (ES) and energy expenditure (EE). AU, arbitrary unit; WT, wild type; TAC, transverse aortic constriction; MCFA, medium-chain FA. + basal, ++ increased, → basal, ↓ reduced, ➘ mildly reduced.

It was recently reported that pool size in the TCA cycle is a useful marker for energy status in the heart in vivo [47,50,66,67]. Although pool size is not directly linked to cardiac contractile function in animal models per se, it becomes more useful to assess energy status in combination with the concept of energy supply (ES) relative to energy expenditure (EE). Figure 3 shows a proposed model of pool size that reflects the net effect of wall stress, contractility, heart rate, and the difference between ES and EE.

EE in the heart is minimal at rest and positively associated with an increases in heart rate, wall stress, and contractility [68]. In the hearts of mice with reduced FA uptake, ES was found to be lower than that in wild-type (WT) hearts, which caused a reduced pool size in the TCA cycle. However, the reduced ES was found to be sufficient for basal cardiac function because the required EE was also small. When the EE is elevated by an increased workload, such as transverse aortic constriction (TAC), the ES is simultaneously enhanced to meet energy demand in the WT heart. However, in the hearts of mice with reduced FA uptake, limited FA uptake was shown to result in diminished ES, leading to reduced EE compared to WT-TAC hearts. Because wall stress is elevated by TAC, reduced EE is directly linked to reductions in contractility. When an alternative energy substrate, such as medium-chain FAs (MCFAs), is supplemented, the ES is enhanced, which increases the EE and contractility but not pool size. Because it is technically difficult to precisely measure high-energy phosphate due to its instability and pool size is more sensitive than high energy phosphate, pool size could be used an alternative marker to assess the energy status of the heart. Further studies are needed to prove this hypothesis.

### 4.6. Mechanism Underlying the Enhancement of Glycolytic Flux in the Hearts of Mice with Reduced Fatty Acid Uptake

Although the precise mechanism underlying a compensatory increase in glucose uptake in the heart with reduced FA uptake has not been determined, the glucose–fatty acid cycle (the so-called Randle cycle) likely plays a significant role. In this concept, the heart prefers long-chain FAs as the primary fuel, and increased intermediates of FA oxidation restrict glucose metabolism via allosteric inhibition [69]. The allosteric inhibition of several glycolytic steps, such as hexokinase (HK) and phosphofructo-1-kinase (PFK-1), is mediated by citrate in the cytosol, whereas pyruvate dehydrogenase (PDH) inhibition results from the accumulation of acetyl-CoA and NADH. As described above, in hearts with reduced FA uptake, limited FA use was found to cause a reduction in the pool size (total intermediates) in the TCA cycle [47,50], which could result in accelerated glycolysis. Even in a streptozotocin (STZ)-induced type I diabetes model, a compensatory increase in glucose uptake was not suppressed [66], which strongly suggests that enhanced glucose uptake is independent of insulin and the insulin-induced translocation of GLUT4, but it does depend on energy insufficiency. In mice with an inducible deletion of Rbp-jκ in the endothelium, the expression of Glut4 was found to be reduced, but glucose uptake was rather elevated [39], which further suggests that energy insufficiency by reduced FA uptake, but not GLUT4 induction, is the primary driving force for enhanced glucose uptake. Thus, the compensatory glucose uptake in hearts with reduced FA uptake is likely to be accounted for, at least in part, by the glucose–fatty acid cycle (or Randle cycle).

### 4.7. In Vivo Contractile Dysfunction in Mice with Reduced Trans-Endothelial Fatty Acid Transport in Streptozotocin-Induced Diabetic Cardiomyopathy

Diabetic cardiomyopathy occurs in patients with diabetes independently of other cardiovascular risk factors, including hypertension and ischemic heart disease [70,71]. Although diabetic cardiomyopathy is induced by multifactorial mechanisms [72,73,74,75,76], the lipotoxicity is a key event that drives cardiac dysfunction in diabetes. Excessive FAs are converted into other lipid intermediates with lipotoxic effects, such as ceramides. Increased levels of ceramides can stimulate inflammatory signaling and facilitate the production of reactive oxygen species (ROS), which consequently results in the impairment of mitochondrial energetics. However, whether reduced FA uptake is protective against lipotoxic effects in diabetic hearts in vivo had been previously unanswered. To address the question, FABP4/5 DKO and CD36 KO mice were employed to evaluate the contribution of a reduction in FA uptake to the development of diabetic cardiomyopathy generated by STZ treatment [66,77]. Contrary to initial expectations, cardiac contractile dysfunction by STZ was exacerbated in the FABP4/5 DKO and CD36 KO mice. Although compensatory glucose uptake was maintained, the glycolytic flux into the TCA cycle was strongly suppressed. The total energy supply, estimated with pool size in the TCA cycle, was also reduced in the FABP4/5 DKO and CD36 KO hearts. The levels of ceramides in the hearts were comparable to those in the WT hearts or reduced. Thus, enhanced FA uptake in diabetic hearts seems to be a compensatory response to a reduced energy supply from glucose, so limited FA use could be detrimental to contractile function due to energy insufficiency. More in vivo experiments are needed to verify this notion.

## 5. Conclusions

Muscle-type continuous capillaries regulate TEFA transport, a rate-limiting step in FA uptake by parenchymal cells, including cardiomyocytes. The transport of FA across the capillary endothelium comprises three steps—lipolysis on the luminal side of the endothelium, FA uptake by the plasma membrane, and intracellular FA transport by cytosolic proteins (Figure 1). Molecules involved in TEFA transport are relatively specific to the capillary endothelium in the heart and skeletal muscle, which has been shown by independent transcriptome analyses [78,79]. TEFA transport is regulated by multiple transcription factors and signaling pathways (Table 1), some of which are influenced by nutritional status. Although the defective function of TEFA transport results in reduced FA uptake by the heart, mice exhibit preserved cardiac function under unstressed conditions in most cases, probably due to the local and systemic compensation of other fuels such as glucose and ketone bodies (Table 2). When the workload is increased or gene deletion is induced at adulthood, energy balance by compensatory mechanisms collapses in the heart, leading to contractile dysfunction (Figure 2). The supplementation of alternative fuels via the ingestion of an MCFA-rich and ketogenic diet has been found to restore contractile dysfunction, at least partially, indicating that energy insufficiency due to reduced FA supply is the predominant cause of cardiac dysfunction. Thus, FA is a central energy substrate in the heart, even under diseased conditions, and the capillary endothelium is a critical component in the heart for sufficiently supplying FA to the cardiomyocytes. Since a reduced FA oxidation capacity causes energy starvation in a failing heart, modulating the technology of TEFA transport via several possible pathways, including PPARγ, Notch, and Meox2/TCF15, might open a new avenue to developing therapeutic strategies for energy-starved heart failure.

Most of the findings on cardiac metabolism introduced in this review were obtained by recent in vivo experiments, which sometimes opposed previous concepts established by ex vivo and in vitro observations. Inconsistent results between in vivo and in vitro studies on metabolism have also been reported in other fields, such as cancer biology [80]. A recent advancement in metabolic research is in vivo flux analysis with multiple stable isotopes, which provides opportunities to reveal unappreciated processes of metabolism in the context of whole animals [5,81,82,83,84]. Accordingly, it is warranted to re-estimate the functional significance of each substrate in diseased hearts in vivo with significantly refined technologies, which will further aid our understanding of the pathophysiological association between cardiac dysfunction and metabolic adaptation/maladaptation.

## Data Availability

The data presented in this study are available in article.
